# Squaring the Circle. Social and Environmental Implications of Pre-Pottery Neolithic Building Technology at Tell Qarassa (South Syria)

**DOI:** 10.1371/journal.pone.0042109

**Published:** 2012-07-27

**Authors:** Andrea L. Balbo, Eneko Iriarte, Amaia Arranz, Lydia Zapata, Carla Lancelotti, Marco Madella, Luis Teira, Miguel Jiménez, Frank Braemer, Juan José Ibáñez

**Affiliations:** 1 Departamento de Arqueología y Antropología, Istitució Milà i Fontanals, Consejo Superior de Investigaciones Científicas (IMF-CSIC), Barcelona, Spain; 2 Departamento de Ciencias Históricas y Geografía, Universidad de Burgos, Burgos, Spain; 3 Geografia, Prehistoria eta Arkeologia Saila, University of the Basque Country (UPV/EHU), Vitoria-Gasteiz, Spain; 4 Instituto de Historia, Centro de Ciencias Humanas y Sociales, Consejo Superior de Investigaciones Científicas (CCHS-CSIC), Madrid, Spain; 5 Istituto Català de Recerca i Estudis Avançats (ICREA) at IMF-CSIC^(a)^, Barcelona, Spain; 6 Instituto Internacional de Investigaciones Prehistóricas de Cantabria (IIIPC), Santander, Spain; 7 Donato arquitectos asociados, Barcelona, Spain; 8 Culture et Environnement Préhistoire Antiquité Moyen Age, Centre National de la Recherche Scientifique (CEPAM -CNRS), Université de Nice, Nice, France; University College London, United Kingdom

## Abstract

We present the results of the microstratigraphic, phytolith and wood charcoal study of the remains of a 10.5 ka roof. The roof is part of a building excavated at Tell Qarassa (South Syria), assigned to the Pre-Pottery Neolithic B period (PPNB). The Pre-Pottery Neolithic (PPN) period in the Levant coincides with the emergence of farming. This fundamental change in subsistence strategy implied the shift from mobile to settled aggregated life, and from tents and huts to hard buildings. As settled life spread across the Levant, a generalised transition from round to square buildings occurred, that is a trademark of the PPNB period. The study of these buildings is fundamental for the understanding of the ever-stronger reciprocal socio-ecological relationship humans developed with the local environment since the introduction of sedentism and domestication. Descriptions of buildings in PPN archaeological contexts are usually restricted to the macroscopic observation of wooden elements (posts and beams) and mineral components (daub, plaster and stone elements). Reconstructions of microscopic and organic components are frequently based on ethnographic analogy. The direct study of macroscopic and microscopic, organic and mineral, building components performed at Tell Qarassa provides new insights on building conception, maintenance, use and destruction. These elements reflect new emerging paradigms in the relationship between Neolithic societies and the environment. A square building was possibly covered here with a radial roof, providing a glance into a topologic shift in the conception and understanding of volumes, from round-based to square-based geometries. Macroscopic and microscopic roof components indicate buildings were conceived for year-round residence rather than seasonal mobility. This implied performing maintenance and restoration of partially damaged buildings, as well as their adaptation to seasonal variability.

## Introduction

The Pre-Pottery Neolithic (PPN) period is crucial to the understanding of a major step in human history involving the emergence of farming and the shift to sedentism. In the Levant, the process of animal and plant domestication crystallizes in the 9^th^ millennium BC and is associated to the second phase of the PPN (PPNB). With domestication, aggregated settled life in clustered settlements also became a generalized phenomenon [1]. The study of PPN architecture has revealed key aspects of the social complexity that characterised early farming communities [2], [3]. Previous studies have focused on the function of the buildings (residential, storage, ritual). Instead, our focus on early architecture technology aims at exploring the social and environmental implications of the transition from mobile to sedentary life. In this sense, information on building conception, construction, maintenance (rebuilding, reparation) and destruction, is interpreted as a proxy of human socio-ecological behaviour. Evidence is drawn from high-resolution direct observation of macroscopic and microscopic remains of mineral and organic building components from a PPNB building found at Tell Qarassa.

Extensive reviews of Late Pleistocene and Early Holocene building typologies from the Near East (mostly Natufian and PPN) have been previously outlined [1], [4]. Two main tendencies emerge: **(a)** the shift from round to square structures, **(b)** the tendency towards ever more complex buildings; i.e. from isolated to clustered, from single- to multiple-room, from single- to multiple-storey [5]. Round buildings have been found in pre-Neolithic (Natufian) [1], [4] and in early PPN (PPNA) contexts across the Levant. In the Middle Euphrates (North Levant), Tell Mureybet [3], [6] and Jerf el Ahmar [3], [7] have provided PPNA transitional architectural contexts, where round and squared buildings have been found in the same stratigraphic levels. The PPNA levels at Jerf el Ahmar have also given evidence of transitional buildings, square with round corners [3], [7]. The generalized shift towards squared buildings is a trademark of the PPNB, when this typology becomes common across the Levant, as at Beidha [4], [8] and ‘Ain Ghazal [9], in the South. In Central and South Levant the transition from round to square architecture is associated to the Early PPNB (EPPNB), e.g. at Motza [10]. However, evidence of EPPNB architecture is scarce in the region [11], [12], where the transition from round to square typologies remains poorly understood.

The construction of hard buildings is associated to the generalized use of stone, wood, mud and daub [13]–[16]. Buildings in PPN contexts have commonly been described at the macroscopic level, focusing on building geometry (perimetral walls), mineral components (e.g. daub, plaster and stone) and large wooden elements. Interpretations on the use of wooden and other plant components is often based on their arrangement and taxonomical identification, or depend largely on ethnographic analogy (e.g. Rajif Village, South Jordan [5]) and tends not to go beyond the macroscopic level even when preservation conditions are favourable (e.g. Ganj Dareh Tepe [17]). These trends result from the concurrency of critical excavation conditions in rescue archaeology contexts (e.g. Tell Mureybet and Jerf el Ahmar [3], [7], ‘Ain Ghazal [18]), and adverse preservation circumstances (e.g. Çayönü Tepesi [19]). In some cases, the study of buildings has involved laboratory and microscopy-based analyses. For example, micromorphology has been used to evaluate site formation processes at several Neolithic and Bronze Age urban sites in the Near East [20], [21]. Physico-chemical characterization has been used in Neolithic contexts at Çatalhöyük to extract social information from mudbrick compositions [22]. Microscopy has been performed on most organic remains from the Palaeolithic huts of Ohalo II [23], [24]. Microscopic identification of silica skeleton remains (phytoliths) has been done at several Near East Neolithic contexts [25].

Few examples of direct archaeological observation of roof components stand out within the PPN period. A flat roof made of wooden beams, with superposed wooden planks and topped with daub has been described from the PPNA levels at Jerf el Ahmar [7], where the use of plant materials other than wood has been recorded through the study of impressions of wild cereal chaff used as temper in daub and mud walls [26], [27]. A flat roof made of wood, branches and mud has been described within Middle PPNB contexts at Shkârat Msaied [5]. The detailed reconstruction of a similar roof has been proposed for the Late Pre-Pottery Neolithic B (LPPNB) levels of Ba’ja, although entirely based on field macroscopic observations [28].

**Figure 1:**
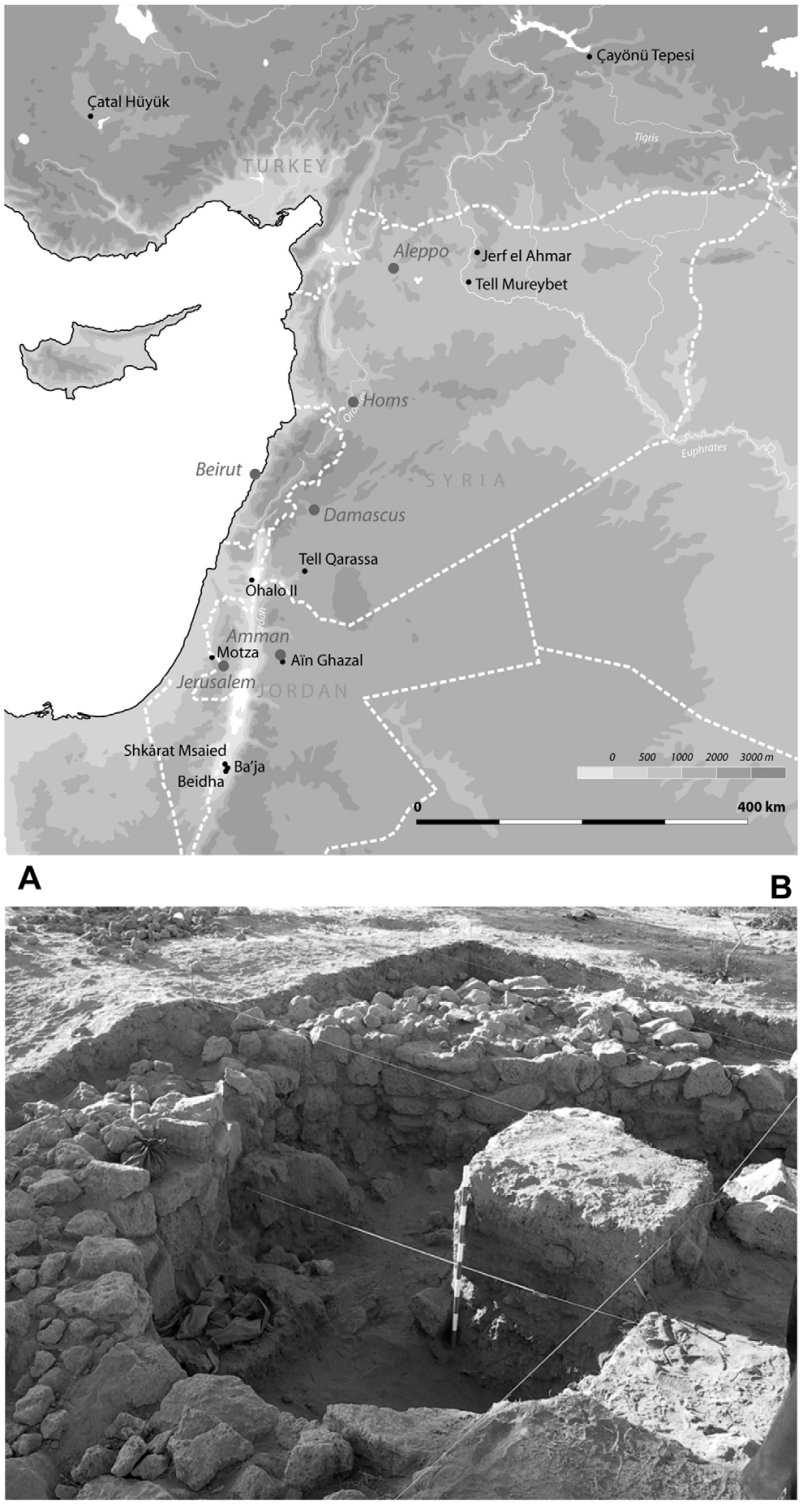
Location map and site view. (A) Location map indicating archaeological sites cited in the text, with the exception of Ganj Dareh Tepe (Iran) located outside the frame. (B) Photo showing the reference sediment column at the centre of the building before excavation.

EPPNB structures at Qarassa were discovered on the North tell in 2007. Architecture can be defined as transitional, showing clusters of rectangular buildings with round corners (Fig. 1). Extensive excavations started in 2009. In 2010 the remains of a collapsed burned roof were excavated. Tell Qarassa is a unique case for Central and South Levant in terms of: **(a)** chronology (EPPNB), **(b)** geography (central Levant) and **(c)** applied integrated methodologies (microstratigraphy, phytolith and wood charcoal analyses). Derived taxonomical and taphonomical information takes us to a sharper definition and deeper understanding of the environmental, social and behavioural implications of buildings technology and settled life within the framework of the domestication process in the early Holocene of the Levant. In these terms, this study provides a new insight into the capability early sedentary communities had to manage the environment within which they lived.

## Results

### Site Stratigraphy

**Figure 2:**
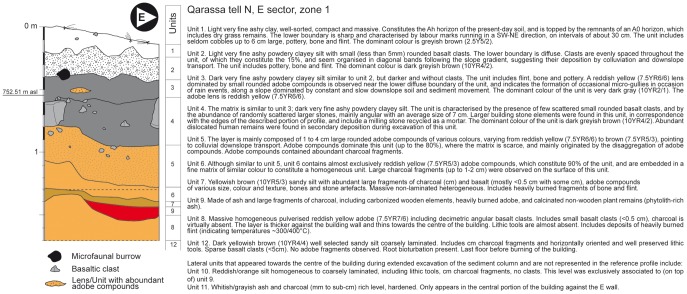
Site stratigraphy. Description of the reference stratigraphic column for Qarassa Tell North Zone 1.

The upper portion of the South-facing profile of the sediment column situated in the centre of the building was taken as reference and described and sampled in detail (Fig. 2). Results described below focus on unit 9 (collapsed burned roof).

### Size and Disposition of Roof Elements

**Figure 3:**
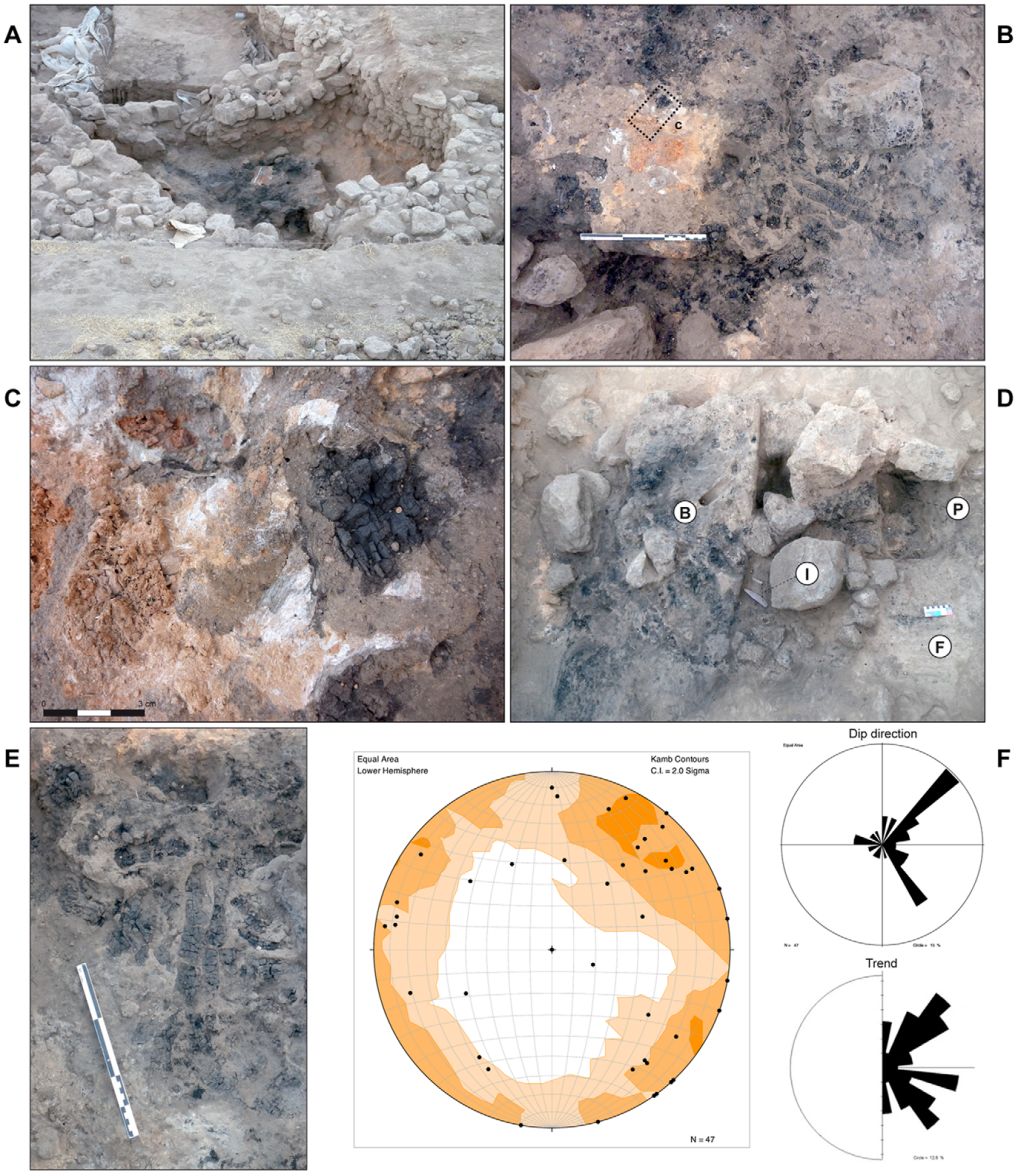
Burned roof unit (unit 9). (A) Overview of the excavated building. (B) Burned roof elements inside the building with position of sample for thin section production. (C) Detail of (B) showing the three elements of the burned roof: burned beams, white phytolith ash layer and reddened daub layer. (D) Architectural elements within and under the burned roof: F: Floor, P: Posthole, I: Flint tools. B: wooden roof elements. (E) Detail of burned beams from the wooden structure of the roof. (F) Stereographic projection of trend and plunge for all recorded burned beams and rose diagrams showing dip and trend for recorded burned beams.

A partial structural reconstruction of the roof is proposed based on the recording of position, width, length and orientation of the majority of the excavated burned beams (BB) (Fig. 3). Overall, burned beams cluster into two almost perpendicularly oriented groups (c. 20 units per group), with a third group emerging between them (Fig. 3 F). There is no visually obvious correlation between beam size and orientation (i.e. the widths of the beams seem to be randomly distributed relative to their orientation). Likewise, there is no apparent correlation between beam size, orientation and taxon (i.e. wood types seem to have been used without particular discrimination as to the constituents of the roofing). Burned beam widths are included between 20 and 100 mm, with the exception of BB49 (identified as a post) that has a width of 140 mm and was originally inserted into the floor of the building to a depth of c. 300 mm. The posthole within which the post was inserted has a cross-section diameter of 200 mm.

### Wood Charcoal Analysis and Radiocarbon Dating

**Figure 4:**
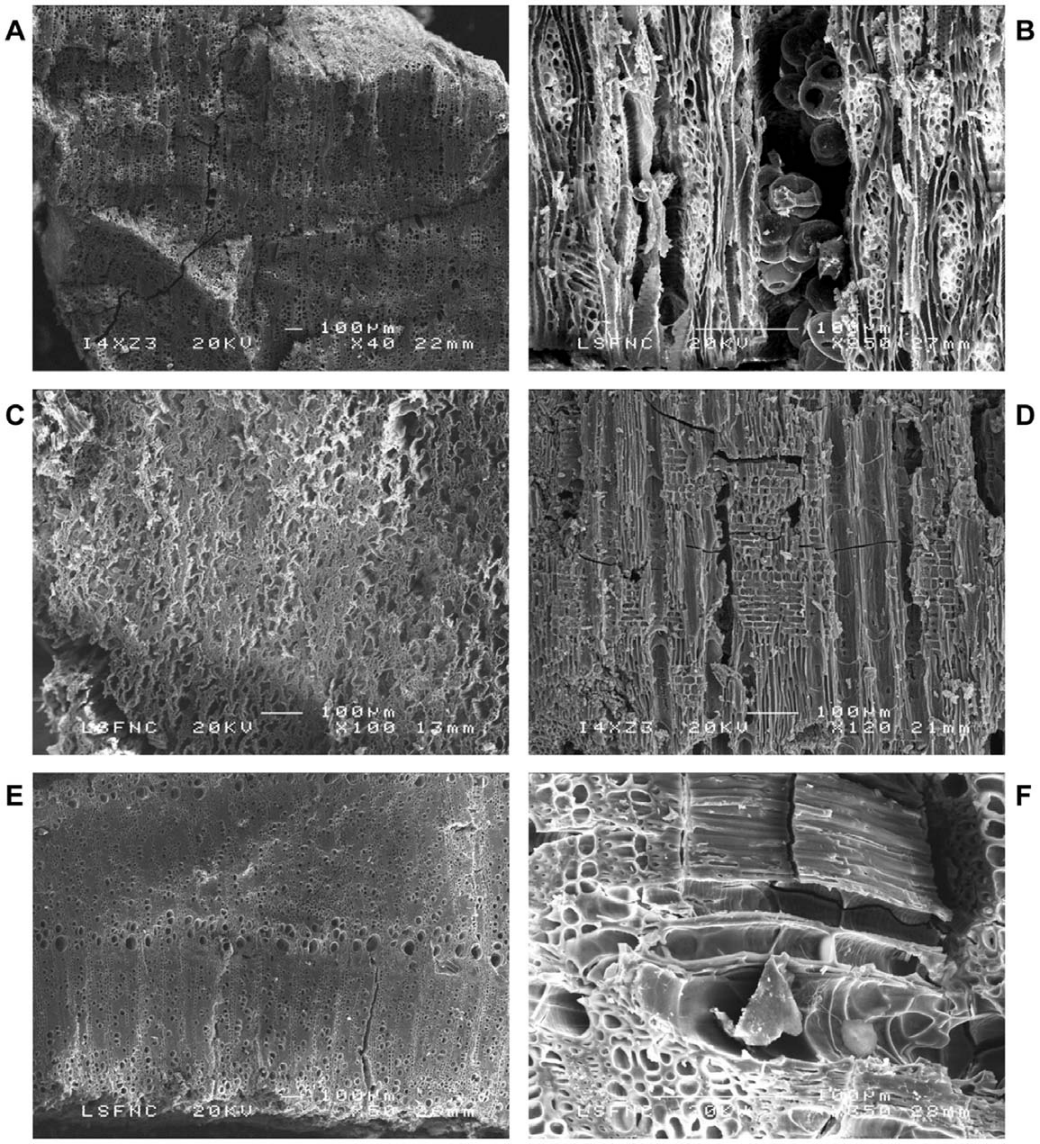
SEM photographs of wood charcoal. (A) *Pistacia* sp. (BB18) transversal section. (B) *Pistacia* sp. longitudinal-tangential section (BB 42) showing a gallery with unidentified round-shaped remains. (C) Salicaceae cf. *Salix* (BB46) showing deformed transversal section. (D) Salicaceae cf. *Salix* (BB3) radial section showing heterogeneous rays. (E) *Amygdalus* sp. (BB 10) transversal section and (F) radial section with hiphae remains.

All burned beams recorded in the field underwent taxonomical characterization (Table 1). Out of the 50 burned elements recorded, 49 were identified as roof beams, and one as a post (BB49). *Pistacia* sp. is the dominant taxon observed. These elements were obtained from deciduous pistachio *Pistacia palaestina* Boiss., *P. atlantica* Desf. or *P. terebinthus* L., rather than evergreen *P. lentiscus* L., as indicated by the presence of ring-porous, distinct growth rings, rays 1–4 cells wide and one row of wide pores at the beginning of the growth ring [29]. The second main taxon is Salicaceae, not presently found in the vicinity of the site (Fig. 4 A and B), although in 1953 Mouterde mentions *Populus bolleana* Mast. in Qanavat and possibly *Salix alba* L. in Kafer, both less than 20 km from Qarassa [30]. Differentiation between *Populus* and *Salix* is not straightforward. Salicaceae have been identified as cf. *Salix* in most cases. The recurrent presence of heterogeneous rays makes the willow the most probable taxon used to obtain these roof elements. *Amygdalus* sp. is recorded in three instances (Fig. 4 C). Identification of the cutting period of the wooden elements was possible in four instances: BB15, BB35 and BB48 (*Pistacia* sp.), as well as BB10 (*Amygdalus* sp.). Cutting period of the trees was spring in all cases since only the early wood of the last ring is present.

**Table 1:** Taxonomic identification of burned beams.

BB n	Length (mm)	Witdh (mm)	Width +43% (mm)	Plunge (°)	Trend (°)	Taxon
BB30	50	50	72	8	42	cf. *Pistacia* sp.
BB1	400	75	107	18	140	Salicaceae cf. *Salix/Pistacia* sp.
BB4		65	93	32	50	*Pistacia* sp.
BB11	400	90	129	10	60	*Pistacia* sp.
BB12		40	57	90	90	*Pistacia* sp.
BB15	80	50	72	0	145	*Pistacia* sp.
BB18	140	60	86	14	60	*Pistacia* sp.
BB22		35	50	0	80	*Pistacia* sp.
BB23		60	86	10	360	*Pistacia* sp.
BB24	140	20	29	0	138	*Pistacia* sp.
BB27	110	40	57	0	190	*Pistacia* sp.
BB31	30	20	29	6	26	*Pistacia* sp.
BB32	25	27	39	50	36	*Pistacia* sp.
BB33	40	40	57	48	8	*Pistacia* sp.
BB34	170	45	64	20	56	*Pistacia* sp.
BB35	180	50	72	10	287	*Pistacia* sp.
BB36	60	30	43	40	310	*Pistacia* sp.
BB38	200	100	143	0	137	*Pistacia* sp.
BB39	210	75	107	70	110	*Pistacia* sp.
BB40	140	40	57	26	40	*Pistacia* sp.
BB41	60	40	57	20	40	*Pistacia* sp.
BB42	100	70	100	0	165	*Pistacia* sp.
BB43	100	35	50	0	144	*Pistacia* sp.
BB44	40	40	57	45	243	*Pistacia* sp.
BB47	320	100	143	15	2	*Pistacia* sp.
BB48	200	35	50	6	278	*Pistacia* sp.
BB49	100	140	200	16	22	*Pistacia* sp.
BB51	140	65	93	12	279	*Pistacia* sp.
BB52	400	95	136	10	306	*Pistacia* sp.
BB53	300	70	100	46	335	*Pistacia* sp.
BB10	140	50	72	38	40	*Amygdalus* sp.
BB54						*Pistacia* sp.
BB55						*Pistacia* sp.
BB28	100	30	43	12	282	*Amygdalus* sp.
BB29	120	25	36	20	52	*Amygdalus* sp.
BB2	450	70	100	0	170	Salicaceae
BB3	140	75	107	20	146	Salicaceae cf. *Salix*
BB20	120	50	72	0	100	Salicaceae/*Pistacia* sp.
BB13	170	28	40	18	253	Salicaceae cf. *Salix*
BB16	250	35	50	0	40	Salicaceae cf. *Salix*
BB17	330	30	43	0	40	Salicaceae cf. *Salix*
BB19	270	70	100	20	140	Salicaceae cf. *Salix*
BB21	70	45	64	10	360	Salicaceae cf. *Salix*
BB25	150	60	86	16	125	Salicaceae cf. *Salix*
BB26		60	86	0	110	Salicaceae cf. *Salix*
BB37	170	100	143	0	70	Salicaceae cf. *Salix*
BB45	60	35	50	25	208	Salicaceae cf. *Salix*
BB46	150	40	57	28	214	Salicaceae cf. *Salix*
BB50	110	100	143	35	124	Salicaceae cf. *Salix*
BB14						Salicaceae cf. *Salix*

BB48 provided material for AMS radiocarbon dating, BB49 is the central post.

Radiocarbon dating was obtained from a thin branch (BB48). An additional date was obtained from a charred seed (*T. monococcum* L., wild/domestic einkorn) extracted from the upper portion of the original floor (unit 12 in Fig. 2), representing the last occupation phase of the building before abandonment and roof collapse (Table 2). The ages obtained from the roof elements (BB48) and the floor (charred seed) were performed at different laboratories and provide consistent results suggesting a probable age for the use of the building 10.5 ka BP. This places the building within the Early PPNB period [1].

**Table 2:** Calibrated radiocarbon ages.

Lab code	Sample name andmaterial	14C age uncal BP	Cal BP 1 sigma	Cal BP 2 sigma	Cal BC/AD 1 sigma	Cal BC/AD 2 sigma	Median cal BP	Median BC/AD
CNA - 1065	Charred branch BB48 (*Pistacia* sp.)	9300±45	10426–10466 (0.27)10481–10573 (0.73)	10289–10331 (0.04)10338–10356 (0.02)10371–10599 (0.91)10624–10650 (0.03)	8624–8532 (0.73)8517–8477 (0.27)	8701–8675 (0.03)8650–8422 (0.91)8407–8389 (0.02)8382–8349 (0.04)	10501	8551
Beta –290929	Charred seed (*Triticum monococcum* sp.)	9340±40	10502–10589 (0.88)10629–10647 (0.12)	10426–10466 (0.07)10481–10679 (0.93)	8698–8680 (0.12)8640–8553 (0.88)	8730–8532 (0.93)8517–8477 (0.07)	10555	8605

### Wood Alterations

Four main types of alteration were observed during anthracological analysis: **(a)** xylophagous galleries in many charcoal fragments from *Pistacia* sp. and Salicaceae. Some fragments have more galleries than others. Galleries are of different size (0.14 to 3.70 mm) and shape (round to oval), possibly corresponding to different insect types. Some galleries are filled with insect excrements made of plant tissue, **(b)** microscopic fungal mycelia filaments in at least eight beams, **(c)** modified cell patterns in at least 13 beams including cell deformations from intact cells to complete destruction of secondary cell structure. Several *Salix* sp. beams present a deformed cell pattern comparable to the “wavy” deformation left by brown-rot fungus, commonly associated with wooden structures ([31] and references therein), **(d)** vitrification in at least one instance (BB41). It has been suggested that vitrification may result from a combination of factors including the use of green wood and burning at high temperatures in reduction atmosphere [32]–[34].

Wood elements cut in spring present different degrees of alteration. BB10 and BB48 have a low degree of alteration with few xylophagous galleries and fungal mycelia filaments. In contrast, most cells in BB15 are filled with fungal mycelia filaments and BB35 has extensive evidence of xylophagous galleries of different size.

### Microstratigraphy and Micromorphology

**Figure 5:**
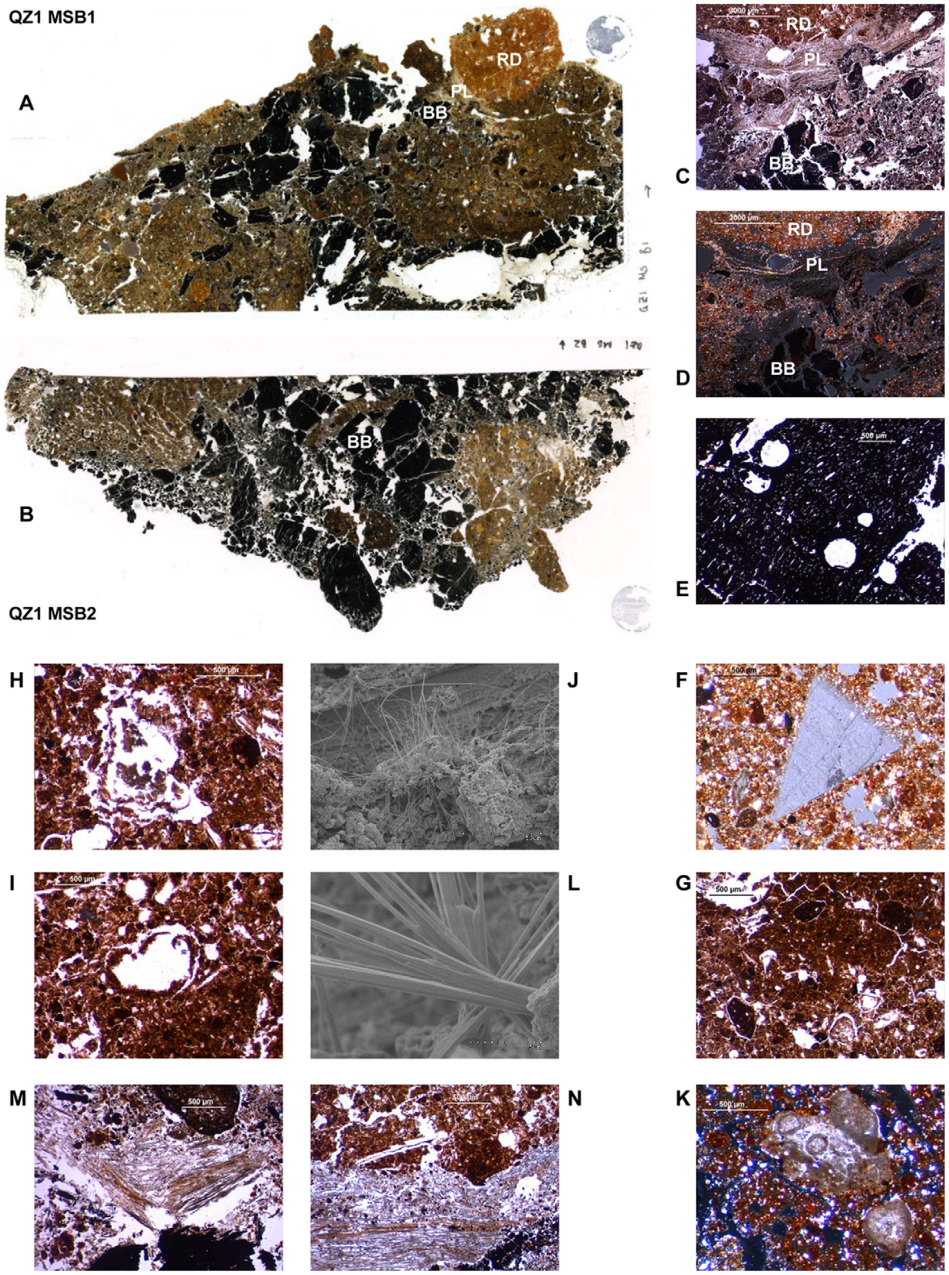
Micromorphological features from unit 9. (A) Thin section QZ1MSB1 (125 mm across horizontally). (B) Thin section QZ1MSB2 (125 mm across horizontally). (C) Detail of interconnected microfacies PPL. (D) Detail of interconnected microfacies XPL. (E) Pistachia burned beams in thin section with parasite channels. (F) Fragments of lithic material. (G) OM-rich sediment aggregates. (H) Voids with preserved ash pseudomorphs of plant cellular structures. (I) Voids with unburnt dehydrated plant remains and/or parenchimatic amorphous matter. (J and L) Voids showing plant impressions from grass culm/leaf filled with needle-fiber calcite visible on SEM imaging. (K) Ash compounds. (M) Two superposed bands of phytoliths. (N) Neat fracture within the phytolith bands.

Micromorphological samples were taken from unit 9 representing the roof collapse (Fig. 2 and 3 B). Observations were made on two thin sections: QZ1MSB1 and QZ1MSB2 (Fig. 5 A–D). Three superposed microstratigraphic units were observed: **(a)** (bottom) groundmass containing sectioned burned beams (BB), **(b)** (centre) phytolith-rich ash lense (PL) and **3** (top) reddened daub (RD).

The reddened daub presents a very heterogeneous fabric including void pseudomorphs after plant remains and plant tissue shrinkage voids [35]. The observed void pseudomorphs may be subdivided into four groups: **(a)** “clean voids” (empty), **(b)** “ash pseudomorph voids” filled with preserved ash pseudomorphs of plant cellular structures (Fig. 5 H), **(c)** “parenchimatic voids” filled with unburnt shrinked plant remains and/or parenchimatic tissues (Fig. 5 I) and **(d)** “needle-fibre voids” filled with needle-fibre calcite visible on SEM imaging (Fig. 5 J and L). The daub contains a variety of materials including: amorphous organic matter (OM), ash compounds (Fig. 5 K), organic-matter-rich (OM-rich) sediment aggregates (Fig. 5 G), fragments of lithic material (Fig. 5 F) [36], smaller daub aggregates, bone fragments, charcoal fragments, phytoliths.

### Needle-fibre Calcite

Needle-fibre calcite was observed within void pseudomorphs embedded in the porous daub. Their spatial arrangement has previously been described as a self-supporting random open mesh infilling the centres of voids and root channels (second morphological group in 37). In our case, needle-fibre has a similar self-supported random arrangement but is found on the internal walls of voids left by the decomposition of plant elements embedded within the adobe (mostly leaves and culms). As previously observed, the mineralogy of the needle-fibre calcite included in the daub is virtually pure CaCO_3_ [37].

### Phytoliths

Phytoliths were observed across QZ1MSB1. The main concentration is found within the phytolith-rich ash lens (PL), between the reddened daub (RD, above) and the burned beams (BB, below). Two bands of phytoliths can be distinguished here. **(a)** The upper band is in direct contact with the reddened daub and shows random orientation of the phytoliths, some plant tissue structures (as phytoliths) are observed in cross section. **(b)** Phytoliths in the lower band are articulated (silica skeletons), horizontally oriented and present virtually no fragmentation, plant tissue structures are observed lengthwise (Fig. 5 M). A single neat fracture is observed within this second layer (Fig. 5 N).

### Phytolith Analysis

**Figure 6:**
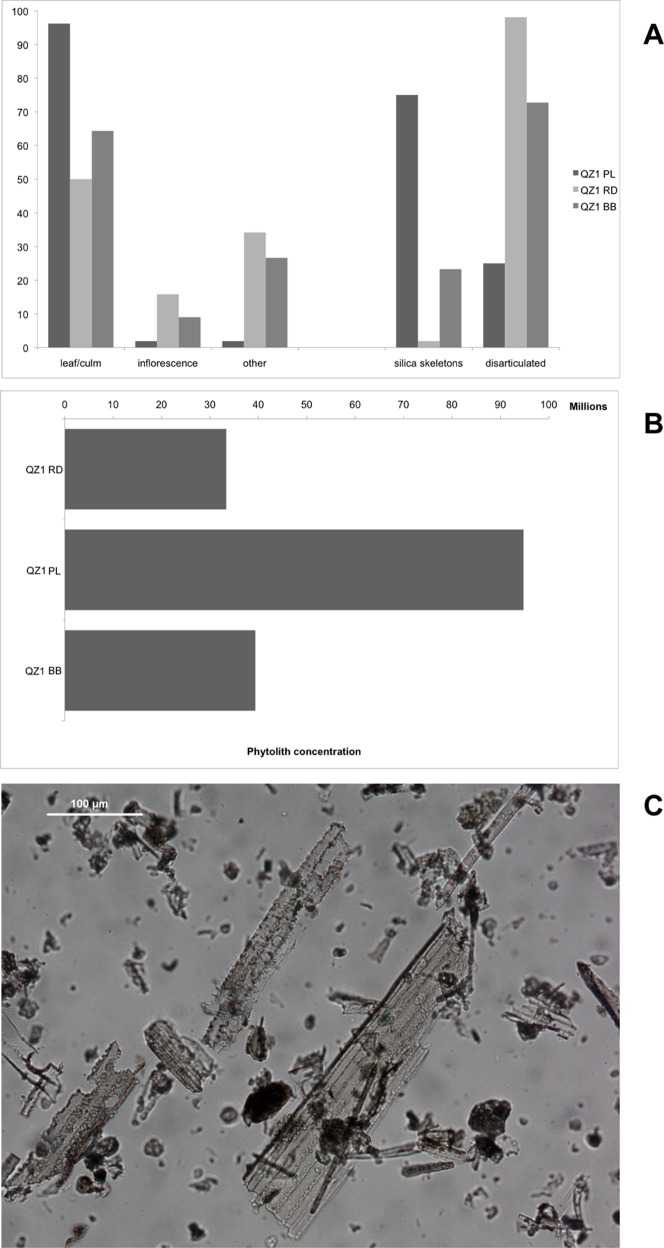
Phytolith concentrations. (A) Phytolith concentrations per gram of AIF. (B) Phytolith morphotype distribution. (C) Detail of phytoliths from PL.

Phytoliths were extracted from three loose samples associated with the roof microstratigraphic units: **(a)** sectioned burned beams (BB), **(b)** phytolith-rich ash lens (PL) and **(c)** reddened daub (RD). Phytolith concentration in unit PL is three times higher than in the other two samples (Fig. 6 A). However, phytolith assemblage composition is similar in the three samples. The majority of morphotypes relates to Pooideae (C3 grasses) leaf and culm (Fig. 6 B). Unit PL displays less variability than units BB and RD, where inflorescences and “other” morphotypes related to various plant tissues are more represented, as well as morphotypes from Cyperaceae leaves (sedges). Units PL and RD show a marked difference in the quantity of silica skeletons (articulated groups of phytoliths) and disarticulated forms (Fig. 6 C), whereas unit BB situates between the two.

## Discussion

Apart from its technological characterization, the study of the building materials used for the construction of the roof at Tell Qarassa has significant social and environmental implications. The shift from round-based (cylindrical and conical) to square-based (cubic) geometries implies a topological shift in the conception and understanding of volumes. Year-round residence, rather than seasonal mobility, implies the long-term maintenance of buildings adapted to seasonal variability. By constructing these new social spaces, PPN communities in the Levant contributed to the creation of new domestic, local and regional environments.

Typological characterization of the building excavated at Tell Qarassa compares with transitional structures, i.e. square buildings with round corners, previously documented in the Near East, e.g. in the PPNA levels at Jerf el Ahmar [3], [7]. This transitional building typology is one of the least documented in the Near East, and Tell Qarassa is the first example of such architecture in Central and South Levant.

The analysed covering structure was made of at least three superposed layers: **(a)** two or more orders of wooden beams resting on the perimeter walls and on at least one central post inserted into the house floor, covered by **(b)** one, possibly two superposed and cross-laid beds of grass leafs and culms, topped with **(c)** porous (light) OM-rich daub. Covering structures made of thick tree branches (c. 50 mm) overlaid with grasses and leaves have previously been attested in the Levant, e.g. in Palaeolithic conical huts at Ohalo II where the topping daub was not present [22], [23].

### A Radial Roof for a Square Building

Although radial roofs have been previously documented for round buildings, e.g. at Tell Mureybet and Jerf el Ahmar [3], [6], [7], [38], Tell Qarassa is the first possible example of a radial covering structure associated with a transitional square building, providing direct evidence of an architectural change that implies a fundamental topological shift for the creation of social spaces.

**Figure 7:**
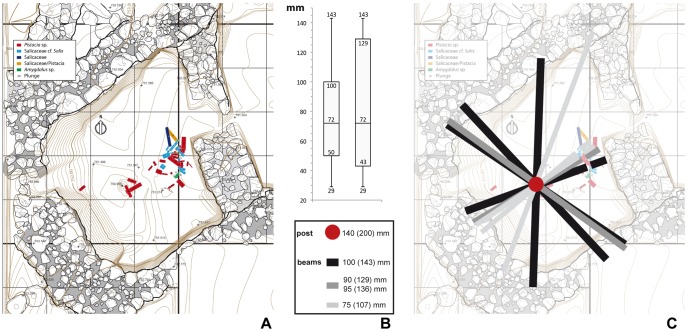
Proposed reconstruction of roof charge structure. (A) Radial distribution of roof beams relative to central post and walls. (B) Boxplot of BBs thickness distribution: in the left boxplot first and third quartiles defines 25% and 50% of the collection, in the right boxplot first and third quartiles defines 13% and 77% of the collection. (C) Proposed radial supportive structure based on (B).

The burned post (BB49) found in the centre of the excavated stone structure (Fig. 7) has a cross-section diameter of 140 mm. It was inserted in a posthole with a cross-section diameter of 200 mm, suggesting shrinkage by burning was at least 43% for building elements made from *Pistacia* sp. A 43% volume loss ratio is derived hereafter to other taxa found at the site.

Supposing that the roof was covered by two orthogonal orders of beams, and that wooden elements were disposed randomly in terms of their thickness (i.e. assuming the roof was covered with beams of mean average thickness 77 mm), the roof would have been structurally viable in terms of self-support, but would not have supported any extra weight on the top. However, when the orientation of large beams (i.e. beams thicker that 129 mm, or above the 85^th^ percentile Fig. 7 B) is plotted on the top of the central posthole, their distribution relative to the post and walls is suggestive of a radial wooden supportive structure for the roof (Fig. 7 C). This pattern is consistent if we extend the supportive structure to beams 107 mm thick (i.e. above the 75^th^ percentile). If we accept that the largest beams were laid radially to support the remaining beams, the roof as a whole would have been able to support several hundreds of kilograms of charge on top of its own weight.

The remaining wooden elements follow two main trends, perpendicular between them and to the walls. They range in size from 20 to 75 mm (i.e. 29 to 107 mm unburnt), and would have generated a network of 150–200 mm wide holes between beams, on the top of the radial supportive structure.

### From Analogy to Direct Observation

Based on microscopic observation, building elements usually derived from ethnographic analogy can be directly studied in primary archaeological context even when preservation conditions are not ideal.

Phytolith morphotypes from the Cyperaceae family (i.e. sedges) are only found within the groundmass of the beams roof unit (BB). Their presence suggests that sedges may have been used to fill the gaps between the network of beams that made the roof supporting structure. Both seed and leaf phytolith morphotypes were observed, indicating that the entire plant was used. Phytolith analysis of the white ash layer (PL) show that Pooideae (C3 grasses) leaves and culms were laid between the wooden structure (below) and the OM-rich daub (above). The primary disposition of phytoliths observed in thin section indicates that two layers of hay or straw were laid perpendicularly on top of each other. This would have permitted hay and straw to support the above daub without the use of twigs, commonly observed in ethnographic contexts ([5] and references therein).

Plant material added as vegetal temper to the overlying daub (RD) has the same origin, although silica skeletons are virtually absent and phytoliths appear mostly disarticulated, attesting their provenance from fragmented grass, e.g. integrated as part of dung and therefore proceeding from chewed fodder. This is consistent with the great variety of ‘recycled’ materials integrated in the topping reddened daub (RD) including fragments of lithic material, bone and charcoal but also daub aggregates and amorphous organic matter.

### A Wetter Environment with Strong Seasonality

Roof technology at Tell Qarassa seems adapted to a climate characterised by strong seasonal variability. Such covering structure constitutes an effective shelter from warm summer temperatures as well as persistent seasonal precipitations.

Wood charcoal analysis of the burned beams suggests local wood was used for the building. The predominant use of *Pistacia* sp. and Salicaceae cf. *Salix* sp. (possibly willow) provides information on the environment surrounding Tell Qarassa. While *Pistacia* sp. is still common in the proximity of the site, willow is presently only found near wet areas. The consistent use of Salicaceae as building elements suggest a more humid environment was present around the site 10.5 ka BP, possibly at the foot of the tell, were a dry depression was filled with water until recent historical periods. This is also suggested by the occurrence of sedge phytoliths within the roof microstratigraphic unit BB. Although adapted to many types of environments, sedges are mainly associated with wetlands and water bodies. In addition, the majority of phytolith morphotypes identified in this and other microstratigraphic units (PL, RD) belongs to the Pooideae grass subfamily that comprises major cereals (e.g. wheat and barley), lawn and pasture grasses. Most species within the Pooideae subfamily are C3 grasses and grow in environments characterized by moderate sunlight and temperatures and abundant groundwater.

Wood parasites and fungal structures were observed in the wooden elements of the roof and in the covering daub. A definitive characterization of observed fungal structures and wood parasites is not possible at this stage, as they cannot be associated directly with any fungi type in absence of fruiting bodies. Nevertheless, Théry-Parisot mentions that the optimal temperature for most fungi is between 24 and 32 C [34]. This provides a plausible hint on average temperatures within and outside built structures in Qarassa at the beginning of the Holocene. Also, the reddened daub mixture used to cover the roof would have dried into a virtually impermeable hardpan once exposed to the sun, constituting an effective shelter in case of sustained seasonal rain, a climatic pattern observed in the Near East from the early phases of the Holocene ([39] and references therein). Such architectural implementations were not attested on the remains of the Palaeolithic huts excavated at Ohalo II [22], [23], occupied during the generally drier and cooler last glacial period ([40] and references therein).

### Lasting Buildings for Sedentary People

Overall, our evidence suggests PPNB buildings at Tell Qarassa were used and sought-after over a long period of time, well beyond that implied by seasonal mobility.

The four types of voids observed within the reddened daub that tops the roof indicate different formation processes. “Clean” and “ash pseudomorph” voids indicate fast disintegration, possibly due to burning. In contrast, “parenchimatic” and “needle-fibre” voids indicate slow decay. “Parenchimatic” voids indicate decomposition possibly initiated within the daub raw material (e.g. dung) before construction and continued until the roof collapse event. Likewise, the formation of needle-fibres seems due to lysis of organic matter by bacteria [37], suggesting that needle-fibres grew slowly along the edges of the voids left by dehydrating leafs and culms used in the original moist daub mixture prior to roof collapse.

Fungal and xylophagous alterations in the wood and daub imply that contamination was previous to the burning of the structure. Besides, altered wood is rarely selected for construction, suggesting that contamination is most likely to have taken place during the use of the building. At least part of the structure must have presented some degree of deterioration at the time of burning. Fungi first decomposes polysaccharides located in secondary cell wall lignin [31], spreading from there through the inter-vascular bordered pits, generating deformed and weakened cell patterns and lagunar areas [41]. As a result, wood becomes weaker with looser consistency. Having lost its consistency, contaminated wood is more prone to combustion, so that accidental burning of the roof structure became more likely as time passed. Some of the analyzed beams were only marginally affected by alterations, suggesting they had been integrated to the structure later than the time of construction, implying that the roof may have undergone some repair. Some of the wooden elements composing the roof were cut in spring, suggesting the structure was constructed in spring/summer. Likewise, roof maintenance work may have taken place in spring/summer, when thin branches and leaves become available.

Some archaeological material was found under the collapsed burned roof: a cluster of flint blades, a possible hoe and some bone fragments. These materials may have been originally placed on the building floor or walls. Their presence suggests that burning may have been accidental. However, the scarce number of findings from the building floor brings to mind cases of buildings left “clean” before intentional destruction, and later conversion into other uses, attested e.g. at Ba’ja [42].

### Conclusions

Our evidence reveals with great detail the different phases and strategies involved in early building architecture. The burned roof excavated within the PPNB levels of Tell Qarassa provides a unique insight into the way that Levantine early Neolithic communities converted resources into buildings adapted to long-term residence in the Near East during the early phases of the Holocene. The presence and use of perishable elements within roofs found in archaeological contexts have often been derived from negative evidence (e.g. post holes for wooden posts) and ethnographic analogy. These approaches have obvious limitations [4]. The present work partly overcomes such limitations providing new results and perspectives derived from the direct observation of microscopic features within roof components of hard-built buildings from the PPNB period.

Tell Qarassa provides one of the earliest attested contexts of transitional PPNB architecture from round to square buildings. A radial wooden support system pivoting on a central post and resting on the perimeter walls, typical of round buildings (e.g. Mureybet), seems to have been adapted here to cover a square building with round corners, hinting to a topological shift in the planning and construction of volumes. Reconstructions of later squared structures found within MPPNB contexts present roofs with supporting structures made of perpendicular beams; e.g. ‘Ain Ghazal [9], [18], Shkârat Msaied [5] and other examples within published reviews [1], [4].

The size of the main support structure at Tell Qarassa suggests the roof had the potential to be used for storage and the passage of people and small domestic animals, something attested for in Near Eastern sites of later periods (e.g. Çatalhöyük [43]). However, deposits found in the layers situated above the collapsed burned roof are mostly made of colluvial and intentionally dumped material (Fig. 2), suggesting that nothing was stored on the roof at the moment of collapse.

Our observations encompass the whole life cycle of the building, including: **(a)** conception of a building derived from composed volumetric concepts, cubic and conical, **(b)** collection in spring of locally found building material for the construction and maintenance of the roof, **(c)** integration of materials in the building shortly after collection, **(d)** long-term use of building materials (year round and probably over several consecutive years) during which parasites could develop, **(e)** creation of cool and moist conditions within buildings, i.e. conditions allowing for the development of fungal and xylophagous alterations, **(f)** destruction by burning of the consistently deteriorated wooden structure, either accidental or intentional.

Buildings adapted to year-round occupation and/or seasonal reoccupation are not exclusive to agricultural groups. For example, seasonal maintenance of brush huts by Palaeolithic hunther-fisher-gatherers at Ohalo II [23] suggests seasonal and year-round occupation took place in the Late Pleistocene on the shores of the Sea of Galilee, set within a highly differentiated ecotone [24]. However, it is with the adoption of agriculture and livestock that the definitive shift from mobile to sedentary life takes place within the Levantine region. Reduced mobility and year-round residence periods meant a new generalised attitude to the local environment, an increasing reciprocity between social and environmental change, less dependent from local ecological traits. The maintenance of sedentism depended on the consistent flow of resources (provided via domestication and foraging) whose success relied in part on socio-ecological strategies suitable to the construction and preservation of the necessary living structures (whether they were residential, storage or ritual). Such strategies included the use of locally available plants for the construction and maintenance of buildings, and the integration of ‘recycled’ materials to obtain buildings suitable for year-round settled life in a region characterised in the Holocene by strong seasonal variability.

## Materials and Methods

### Ethics Statement

All necessary permits were obtained for the described field studies. Permits were obtained from the General Direction of Antiquities and Museums, Damascus. A.R. of Syria: Dr Bassam Jamous General Director, Dr Michel al Maqdissi, Director of Archaeological Excavtions and Research Projects.

### Excavation

The central portion of the PPNB building was chosen during excavation for detailed stratigraphic description and sampling: Munsell chart colour determination and optical characterization (Fig. 2). Remains of carbonized burned beams were exposed in the central part of the building. Individual beams were numbered and their length, orientation and inclination recorded. A thin (millimetric) layer of ash (made of phytoliths) and a centimetric layer of organic matter-rich daub were identified in connection with (superposed) carbonized beams and sampled both loose and as an undisturbed block. Samples were used for physico-chemical and taxonomic analyses and for micromorphology. Discrete individual samples were taken from every carbonized beam, the ash layer, the daub and the groundmass (unit 9 in Fig. 2).

### Wood Charcoal Analysis

Taxa identification was performed on 50 burned beams using incident light microscopy (Olympus BX50) and following standard guidelines and atlases [29], [44], [45] and the reference collection housed at the University of the Basque Country (UPV – EHU). In addition, three flotation samples were taken from the layer including the burnt roof elements. No wood taxa different from those described for the roof elements were identified within this assemblage [46]. Alterations of wood structure and high deterioration of the cell structures observed in some of the burned beams were systematically documented and described.

### Radiocarbon Dating

Radiocarbon dating was performed on short-living elements to avoid old wood effect. Radiocarbon dating was done on the thinnest carbonized BB (branches) from the roof structure and on a seed buried within the underlying floor. Small branches used to build the roof are likely to have been collected immediately before their integration within the roof. Seeds are likely to have been buried within the correspondent archaeological layer shortly after their collection. Radiocarbon ages were calibrated to cal years BP (present is 1950) and years BC/AD using Calib ([47] version 5.0).

### Microstratigraphy and Micromorphology

Scanning electron microscopy (SEM) and optical microscopy were used to analyse undisturbed and discrete microscopic components from elements of the roofing structure. Micromorphological traits were described using a Leica MZ 95 stereomicroscope and a Leica DM 2500. Thin sections were observed under plane-polarized light (PPL) and cross-polarized light (XPL), following established guidelines [48]–[51]. SEM microscopy used was Hitachi S-3500N. Observations focus on general micromorphological traits, taxonomical and taphonomical description of microscopic features and anthropogenic inclusions in building materials. Observed features include: lithic artefacts, bone fragments, ash, plant residues (i.e. parenchimatic organic matter, charcoal, phytoliths), parasite-derived features (xilophagus channels, needle-fibre calcite).

### Phytolith Analysis

Phytoliths were first observed in micromorphological thin section. Three discrete samples extracted from the three microstratigraphic roof units were also observed. Phytoliths were extracted following Madella et al. [52], slightly modified to calculate Acid Insoluble Fraction (AIF), mounted with permanent medium and observed under transmitted light using a Leica DM 2500 microscope. Identification and classification followed established criteria (e.g. [53]) and nomenclature followed the International Code for Phytolith Nomenclature (ICPN) standard [54].
